# Blood management in a patient with anti-Ok^a^ antibody who underwent cardiac surgery using cardiopulmonary bypass: a case report

**DOI:** 10.1186/s12871-020-01120-9

**Published:** 2020-08-20

**Authors:** Yasuhiro Watanabe, Tomofumi Suzuki, Toru Kaneda

**Affiliations:** Department of Anesthesia, Japanese Red Cross Shizuoka Hospital, 8-2 Oute-machi Aoi-ku, Shizuoka, 420-0853 Japan

**Keywords:** Patient blood management, Anti-Ok^a^ antibody, Cardiac surgery, Cardiopulmonary bypass, Frozen thawed red cell

## Abstract

**Background:**

Cardiac surgery under cardiopulmonary bypass (CPB) is often associated with massive bleeding and blood transfusion. For patients requiring specific blood products, meticulous blood management is critical to reduce blood loss, as well as the need for transfusion. Here, we have described the intraoperative blood management in a patient with anti-Ok^a^ antibody, who underwent cardiac surgery with CPB.

**Case presentation:**

A 79-year-old woman was scheduled for open aortic valve replacement and tricuspid valve annuloplasty under hypothermic CPB. Her blood type was A RhD(+) Ok(a−), and anti-Ok^a^, an extremely rare antibody against erythrocyte antigen, was detected. Eight units of Ok(a−) frozen thawed red cells (FTRCs), and six units of red blood cells donated by three Ok(a−) individuals were collected just prior to surgery. Although she was anemic, acute normovolemic hemodilution was conducted after anesthesia induction to preserve the autologous whole blood. Four units of FTRCs were loaded in the CPB priming solution, and modified ultrafiltration was adopted during CPB to prevent further hemodilution. After CPB termination, two units of FTRCs, four units of fresh frozen plasma, and ten units of platelet concentrate were intensively transfused, facilitating surgical hemostasis and stable hemodynamics. The autologous whole blood was returned to the patient in the intensive care unit. Since the hemoglobin and hematocrit levels were maintained postoperatively, no additional transfusion was required throughout her hospital stay.

**Conclusions:**

Multidisciplinary intraoperative blood management in a patient with anti-Ok^a^ antibody facilitated successful cardiac surgery using CPB, along with effective use of limited blood products.

## Background

The Ok blood group system is comprised of Ok^a^, OKGV, and OKVM, all of which are human erythrocyte antigens with high prevalence [1, 2]. Among them, Ok^a^ was first reported by Morel et al. in 1979, after detecting the antibody in the serum of a Japanese female [3]. If anti-Ok^a^ antibodies are produced in the serum of Ok(a−) individuals, extremely rare Ok(a−) red blood cells (RBCs) would be required in the event of transfusion.

Patients undergoing cardiac surgery using cardiopulmonary bypass (CPB) often experience massive bleeding and transfusion due to surgical procedures, dilutional anemia, and the adverse effects of CPB on platelet count and clotting factor activity. Although the probability of requiring blood transfusion during cardiac surgery might be predicted by preoperative risk assessment [4], it is difficult to precisely estimate the amount of blood loss and, therefore, blood products required during surgery. Hence, sufficient amounts of blood products, including RBCs, fresh frozen plasma (FFP), and platelet concentrates (PC) are prepared before cardiopulmonary bypass surgery. However, for patients requiring specific types of blood products, for example, in the present case of a patient with a rare irregular antibody, matched RBCs may not be obtained before surgery. Moreover, blood products may not be available when additional transfusion is required. Therefore, meticulous blood management, to reduce both perioperative blood loss, and the requirement for blood products is of critical importance for such patients.

Here, we describe the intraoperative blood management in a patient with anti-Ok^a^ antibodies, who underwent cardiac surgery with CPB. This case highlighted the importance of a multidisciplinary approach for reducing the requirement of blood products and ensuring effective use of limited resources in cardiac surgery.

## Case presentation

A 79-year-old woman (height, 140.1 cm; body weight, 38.5 kg; body surface area, 1.22 m^2^; EuroSCORE II, 2.93%) with aortic valve stenosis, hypertension, and paroxysmal atrial fibrillation was referred to our hospital. Chest X-ray revealed concentric cardiomegaly. Electrocardiogram demonstrated regular sinus rhythm with 53 beats per min, and complete right bundle branch blockage. Transthoracic echocardiography showed an ejection fraction of 69%, thickening and normal motion of the left ventricular wall, severe aortic valve stenosis, and severe tricuspid valve regurgitation. The aortic valve area, as calculated using the continuity equation, was 0.54 cm^2^, while the peak aortic velocity, and mean pressure gradient were 4.4 m/s and 37 mmHg, respectively. Coronary angiography did not demonstrate any significant stenotic lesions. Medications included amlodipine besylate (2.5 mg), azosemide (30 mg), carvedilol (2.5 mg), and apixaban (5 mg). She was scheduled to undergo surgical aortic valve replacement (AVR) and tricuspid valve annuloplasty (TAP) with MAZE procedure under CPB six weeks later. Her blood type was A RhD(+); however, antibody screening was positive, and the subsequent direct antiglobulin test was negative, indicating the presence of isolated antibodies in the plasma. Consequently, her Ok blood phenotype was revealed to be Ok(a−) and the antibody was anti-Ok^a^.

The preoperative hemoglobin (Hb) levels fluctuated between 10 and 11 g/dL. Furthermore, since Ok(a−) is an extremely rare phenotype, obtaining sufficient amounts of Ok(a−) red blood cell products for the surgery, was a significant concern. From a regional blood center, eight units of frozen thawed red cells (FTRCs) and six units of red blood cells (RBCs) were collected on the day of surgery. The RBCs were donated by three discrete Ok(a−) individuals a few days prior to surgery. Apixaban was stopped two days before surgery. The results of blood tests performed one day before surgery, were not remarkable except for the Hb level being 10.7 g/dL, hematocrit (Hct) 34.4% (Table 1), and N-terminal pro-brain-type natriuretic peptide (NT-pro BNP) 3848 pg/mL (normal, < 55 pg/mL).

**Table 1:**
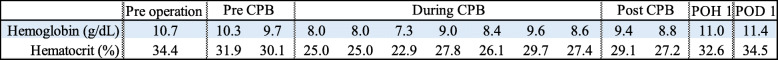
Perioperative hemoglobin (Hb) and hematocrit (Hct) levels. Although the Hct level dropped below 24% once during cardiopulmonary bypass (CPB), the Hb and Hct levels were maintained perioperatively by meticulous blood management

*CPB* Cardiopulmonary bypass, *Hb* Hemoglobin, *Hct* Hematocrit, *POD* Postoperative day, *POH* Postoperative hour

In addition to standard monitoring, regional cerebral oxygen saturation levels (INVOS™ 5100C, Edwards Lifesciences, Irvine, CA, US) were monitored. An arterial catheter was inserted in the radial artery, and remifentanil was infused at 0.4 μg/kg/min, followed by administration of 500 μg fentanyl. General anesthesia was induced with 7 mg midazolam and 40 mg rocuronium. After tracheal intubation, transesophageal echocardiography was performed. A central venous catheter and a pulmonary artery catheter were inserted, and mixed venous oxygen saturation and cardiac output were monitored thereafter (VigilanceII™, Edwards Lifesciences, Irvine, CA, US). Acute normovolemic hemodilution (ANH) was conducted; 400 mL of autologous whole blood was collected via arterial pressure catheter, and 300 mL of crystalloid was rapidly infused. The Hb and Hct levels after ANH were 10.3 g/dL and 31.9%, respectively (Table 1). Anesthesia was maintained with sevoflurane and continuous infusion of remifentanil. Blood pressure and systemic vascular resistance were maintained by norepinephrine (0.02 μg/kg/min) and dopamine (1–3 μg/kg/min) infusion with several boluses of ephedrine (4 mg) and phenylephrine (0.05 mg), until the initiation of CPB. The Hb and Hct levels, immediately after initiating surgery, were 9.7 g/dL and 30.1%, respectively (Table 1), and pre-bypass fluid administration was restricted to 400 mL of crystalloid, including 300 mL for ANH. Tranexamic acid (2000 mg) was administered to suppress hyperfibrinolysis.

The baseline activated coagulation time (ACT) was 158 s. After administration of 12,000 units of heparin, an arterial cannula was inserted into the ascending aorta, followed by cannulation of both the superior and inferior vena cava. CPB, under mild hypothermia, was initiated with spontaneous beating retained. Thereafter, general anesthesia was maintained with a target-controlled-infusion of propofol and an effect-site concentration set at 1.3 μg/mL, along with remifentanil infusion, to maintain the bispectral index (BIS™, Medtronic, Minneapolis, MN, US) between 40 and 60. In advance, four units of FTRCs were loaded in the CPB priming solution (Table 2) to avoid excessive hemodilution, and the Hb and Hct levels immediately after CPB initiation were 8.0 g/dL and 25.0%, respectively (Table 1). ACT was 1000 s, and no additional heparin administration was required during CPB (the shortest value of ACT was 401). After approximately 30 min, body temperature in both bladder and esophagus was stabilized at 30.0 °C. After 1 h from CPB initiation, the Hb and Hct levels decreased to 7.3 g/dL and 22.9%, respectively, and two units of FTRCs were transfused from the CPB circuit, which raised both to 9.0 g/dL and 27.8%, respectively (Table 1). Pulmonary vein isolation and left atrial appendage closure were completed and the ascending aorta was cross-clamped, followed by cardiac arrest that was introduced via antegrade infusion of cardioplegia. After AVR with a biological valve was completed, re-warming was initiated, and TAP was conducted uneventfully. During the valve replacement, cardiac arrest was maintained with intermittent selective and retrograde infusion of cardioplegia. Spontaneous circulation was promptly restored after aortic de-clamping, and CPB was terminated with a dopamine infusion of 5 μg/kg/min. The aortic cross-clamp time was 111 min, and total CPB time was 170 min. Blood tests performed after aortic de-clamping gave the following results: Hb, 8.6 g/dL; Hct, 27.4% (Table 1); platelet count, 12.8 × 10^4^/μL; international normalized ratio of prothrombin time (PT-INR), 1.81; and fibrinogen, 491 mg/dL.

**Table 2:**
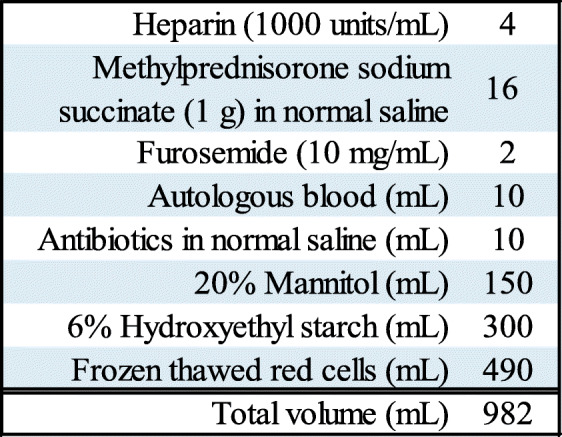
Cardiopulmonary bypass (CPB) priming solution. Four units of frozen thawed red cells (245 mL × 2) were loaded to avoid excessive hemodilution after CPB initiation, and minimal crystalloid was included

*CPB* Cardiopulmonary bypass

After confirming that ACT had returned to 143 s by 120 mg protamine, two units of FTRCs were transfused. In addition, four units of FFP and ten units of PC, as planned prior to surgery, were intensively transfused, facilitating both prompt surgical hemostasis and stable hemodynamics. Oxidized regenerated cellulose (SURGICEL® FIBRILLAR™, Johnson & Johnson, New Brunswick, NJ, US) and human fibrinogen (Beriplast® P Combi-Set Tissue adhesion, CLS Behring, King of Prussia, PA, US) were applied to minimize suture-line oozing of the aorta and right atrium. Minimal bleeding was observed in the surgical field, and 316 mL of salvaged blood, primarily collected from the residual blood in the CPB circuit, was subsequently transfused. Thus, the Hb and Hct levels were maintained above 8.0 g/dL and 27%, respectively, after CPB termination (Table 1). Dopamine infusion was slowly tapered to 1 μg/kg/min, until the end of surgery. The operation duration was 319 min, and total time of anesthesia was 402 min with a total fluid balance of + 546 mL. After the surgery, the patient was transferred to the intensive care unit under intubation, wherein both carbazochrome sodium sulfonate hydrate (25 mg) and tranexamic acid (1000 mg) were administered. Approximately 400 mL of the autologous whole blood was returned to the patient, which raised the Hb and Hct levels to 11.0 g/dL and 32.6%, respectively (Table 1). Patient was extubated at postoperative hour 6, with no neurological deficits. Until postoperative day (POD) 1, blood drainage from pericardium and anterior mediastinum were 80 mL and 50 mL, respectively. The Hct level was consistently maintained above 25%, and thereafter, no additional blood transfusion was conducted throughout the hospital stay. The patient had a good postoperative course, and was discharged on POD 17. We ensured that the six unused units of Ok(a−) RBCs were transfused to other hospitalized Ok(a+) patients within the expiration date.

## Discussion and conclusions

To our knowledge, this is the first case report addressing blood management of a patient with anti-Ok^a^ antibody, undergoing cardiac surgery using CPB. This case highlighted the significance of patient blood management in CPB surgery when the availability of matched blood products is limited.

Although no studies have reported major hemolytic reaction, or disorders, occurring in a fetus and newborn due to the presence of anti-Ok^a^ antibody, the reaction between the anti-Ok^a^ antibody and Ok(a+) RBCs has been reported to reduce red cell survival [3, 5]. Hence, Ok(a−) blood products are preferable for patients bearing the anti-Ok^a^ antibody. An anti-Ok^a^ antibody is generally believed to be produced in the serum of Ok(a−) individuals under two major clinical settings: the delivery of an Ok(a+) newborn, or the receipt of Ok(a+) RBCs by transfusion. Our present case had no history of transfusion; she had, however, delivered two children. Therefore, the anti-Ok^a^ antibody was speculated to have been produced after the delivery. The Ok(a−) phenotype, which does not indicate the total depletion of Ok^a^ protein, but rather the amino acid substitution of glutamic acid at position 92 to lysine [6], is an extremely rare inherited trait worldwide, and has been identified in only eight families in Japan [1]. For patients requiring extremely rare blood products, blood transfusion often relies on FTRCs, which are made from frozen red cells that can be preserved up to 10 years. The blood center of the Japanese Red Cross Society registers individuals with extremely rare blood types, and fortunately, a few individuals with blood type A RhD(+) Ok(a−) were identified. Thus, in addition to the eight units of FTRCs, we were able to obtain six units of Ok(a−) RBCs from the donors, just before the surgery.

Besides other transfusion-related complications such as infection and graft versus host disease, allogeneic blood transfusion (ABT) adversely impacts the patient outcomes associated with cardiac surgery [7, 8]. Therefore, multidisciplinary approaches to reduce the risk of bleeding, and requirement for blood transfusion, is recommended by the recent EACTS/EACTA guidelines [9]. Although preoperative autologous blood donation can reduce RBC transfusion in elective cardiac valve surgery [10], it is not recommended for patients with severe aortic valve stenosis or low Hb levels [9]. Hence, we carefully conducted ANH, which is known to reduce the probability of both ABT and postoperative blood loss in cardiac surgery [11], primarily due to collection of autologous whole blood containing not only red cells but also clotting factors and platelets.

Of note, once the frozen red cells are thawed and transfusable products (i.e. FTRCs) are prepared, they must be transfused within 4 days or else discarded. In addition, the cryopreservation and thawing process of red cells induce mechanical and osmotic stress on red cell surface, hence FTRCs lead to a faster lysis rate than normal RBCs [12]. Alternatively, donated Ok(a−) RBCs can be preserved for 21 days, and transfused to Ok(a+) individuals (vast majority of patients). Therefore, we selected FTRCs over RBCs, and although the patient was anemic, we conducted ANH to facilitate the effective use of both the autologous whole blood, and blood products.

Based on the preoperative estimation of the present case, pre-bypass fluid administration was restricted to 400 mL. Four units of FTRCs were loaded in the CPB priming solution, and minimal crystalloid was included (Table 2). This blood management protocol avoided excessive hemodilution just after CPB initiation. Regarding the blood transfusion threshold in cardiac surgery, recent meta-analysis has compared a restrictive strategy with a liberal transfusion strategy [13]. However, the definite blood transfusion threshold remains unestablished. As to the transfusion management during CPB, current guidelines recommend that packed RBCs should not be transfused if the Hct level is above 24%, and may be transfused based on an assessment of tissue oxygenation for Hct levels between 18 and 24% [14]. In the present case, the hemodynamic status remained relatively stable, with the exception of only one timepoint during CPB (22.9%), the Hct levels were maintained above 25% perioperatively, which may have resulted in avoidance of serious ischemic complications. We also used a cell salvage device (Cell Saver® Elite®, Haemonetics, Braintree, MA, US), and modified ultrafiltration (JMS multi-flow pump MF − 01, JMS, Hiroshima, Japan) in association with the CPB, both of which are recommended to be adopted in cardiac surgery [9]. Modified ultrafiltration has been demonstrated to attenuate the inflammatory response and blood transfusion requirements in cardiac surgery [15]. In fact, ultrafiltration was effective in ameliorating hemodilution during CPB, with the total fluid balance during CPB tightly controlled, resulting in minus 199 mL; however, the Hct levels that dropped below 24% may be attributable to blood loss in the surgical field.

Retrospective analysis of this case revealed other important points regarding patient blood management in cardiac surgery. First, iron and erythropoietin administration to improve anemia can be considered before cardiac surgery. Preoperative blood tests showed normocytic, normochromic anemia, and the Hb and Hct levels were consistently maintained above 10 g/dL and 30%, respectively. Hence, the surgeons had considered the administration unnecessary. Second, using a minimum invasive extra-corporeal circuit to attenuate activation of the coagulation system during CPB, along with point-of-care blood testing (e.g. rotational thromboelastometry), may facilitate more vigilant patient blood management [9], although neither of these is generally available in our hospital. Finally, the patient had an option of transcatheter AVR, which is associated with lower risk of massive bleeding than in surgical AVR [16]. In such cases, the patient would need to be referred to another hospital where the procedure can be conducted.

In conclusion, meticulous intraoperative blood management allowed for successful cardiac surgery and effective use of blood products in a patient with anti-Ok^a^ antibody. Hence, multidisciplinary approaches to reduce blood product requirements are critical for patients with limited availability of matched blood products.

## Data Availability

All relevant data are included in this published article. Other datasets are available from the corresponding author on request.

## Abbreviations

ABT: Allogeneic blood transfusion
ACT: Activated coagulation time
ANH: Acute normovolemic hemodilution
AVR: Aortic valve replacement
CPB: Cardiopulmonary bypass
FFP: Fresh frozen plasma
FTRCs: Frozen thawed red cells
Hb: Hemoglobin
Hct: Hematocrit
NT-pro BNP: N-terminal pro-brain-type natriuretic peptide
PC: Platelet concentrate
POD: Postoperative day
PT-INR: International normalized ratio of prothrombin time
RBCs: Red blood cells
TAP: Tricuspid valve annuloplasty
